# Programming languages in chemistry: a review of HTML5/JavaScript

**DOI:** 10.1186/s13321-019-0331-1

**Published:** 2019-02-05

**Authors:** Kevin J. Theisen

**Affiliations:** iChemLabs, LLC., 7305 Hancock Village Dr #525, Chesterfield, VA 23112 USA

**Keywords:** JavaScript, HTML5, Programming, Chemistry, Cheminformatics, Computational chemistry

## Abstract

This is one part of a series of reviews concerning the application of programming languages in chemistry, edited by Dr. Rajarshi Guha. This article reviews the JavaScript technology as it applies to the chemistry discipline. A discussion of the history, scope and technical details of the programming language is presented.

## Introduction

Computer literacy is an essential skill for scientists. Computers can execute the same operations humans can perform, but far more consistently and at rates far beyond human capability, allowing researchers to investigate numerous hypotheses in short order. Programmers have even more advantages, as they can directly communicate with the computer to achieve their desired goals, as opposed to relying on software someone else has created for another purpose.

Many programming languages have been developed to facilitate instructions to the computer. Each has its advantages, which is why they each exist. Each programming language also has its disadvantages, which is why the rest exist.

When the Internet was introduced, society quickly changed, not just in allowing computers to communicate with each other, but by allowing people to communicate with each other, nearly instantaneously from around the world. JavaScript (JS) is a programming language born of the Internet. From a crude and basic programming language for creating cheap, gimmicky effects on web pages, it has developed into a ubiquitous and flexible technology where engine performance is regarded as a crowning achievement among browser developers. Today, every computational device, from desktops to laptops to mobile phones and Augmented Reality (AR) devices has essential access to the Internet, and therefore contains a web browser wherein JavaScript can be run. As a result, JavaScript is one of the most important programming languages in existence.

This review investigates the relevance and impact of JavaScript on the chemistry discipline. We begin by discussing the history of the programming language; then we investigate the scope and features of the language and how it applies to chemistry; last, an outline of the technical details of the JavaScript programming language is presented to allow those interested in chemistry and cheminformatics to effectively create JavaScript applications.

## What is JavaScript?

JavaScript is a programming language enabling developers to interact with the functionality provided by web browsers. More specifically, JavaScript is a scripting language, which means (a) traditionally, JavaScript source code is interpreted at runtime and not pre-compiled into byte code and (b) practically, its main purpose is to modify the behavior of another application typically written in a different programming language, in which it is interpreted and run in real time.

While JavaScript is aptly named as a scripting language, the first part of the name misleadingly refers to the Java programming language [1]. JavaScript has no functional relationship with the Java programming language. The Java part of JavaScript was used to inspire interest in the new scripting language, as Java was and still is a very popular programming language; additionally the original Netscape browser that included JavaScript was written in Java. JavaScript has since become an essential and arguably more important programming language than the one inspiring its name. Hence, the programming language many developers use today to build and interact with web applications is famously known as JavaScript.

### Origin

In the early days of the Internet, the web browser provided users with static pages to view information. Innovators at Netscape Communications, producers of the popular Netscape Navigator browser, believed web sites should provide more dynamic material and a programming language would provide a solution. In 1995, Brendan Eich, an employee of Netscape Communications, developed what is now known as JavaScript [2].

Initially, JavaScript only provided basic features, but the power of such a programming language on the web was quickly realized, and the continued success of Netscape Navigator was, in no small part, a reflection of that power. Alternatives to JavaScript were inevitable and a number of companies started to produce more feature-rich browser plug-ins that would install their platforms into browsers to be run in web pages. Adobe Flash [3], Java applets [4] and Microsoft Silverlight [5] are a few well known examples. These plug-ins became popular solutions for developers, but a hindrance for users as plug-ins required installations, had to be frequently updated, and were prone to security issues. Plug-ins eventually fell out of favor with users as HTML5 arrived and JavaScript evolved. Flash, Java applets and Silverlight are all now deprecated technologies.

Other companies, instead of replacing JavaScript, attempted to modify and extend the language in an attempt to control it (a process known as “embrace, extend and extinguish”). In 1996, Microsoft produced JScript [6], which was a reverse engineered version of JavaScript for their Internet Explorer (IE) browser. IE would now be accessible to JavaScript developers, but the implementation was deceitful. Proprietary features specific to IE were built into JScript. As a result, users were forced to use IE for many online applications, impeding the open web in a process known as fragmentation. Fragmentation produces an anti-competitive, positive feedback loop giving a dominant group (in this case, the dominant browser, IE) power over the future direction of the Internet. These attempts ultimately failed due to healthy competition amongst browser developers. Microsoft browsers now aim to legitimately support JavaScript. Creative methods for fragmenting the JavaScript language continue to exist with TypeScript from Microsoft [7] and Dart from Google [8], which aim to provide a more familiar programming syntax for developers and compile into standard JavaScript.

JavaScript has survived many challenges since its inception, and a lot of credit should be given to the Netscape team for their foresight. In order to protect the JavaScript language early on, the Netscape team pushed for an open standardization of the language, and they were successful. We all benefit from the universal and strong support for JavaScript in every existing browser today. In spite of the attempts to replace or commandeer the standard, JavaScript persisted and has become a signature programming language for most developers, and remains an important tool for the scientific communities including chemists.

### ECMAScript

The European Computer Manufacturers Association, now known as Ecma International [9], took over the governance and standardization of JavaScript in 1996 and continues to maintain the specification for the language to this day. The JavaScript specification is officially named ECMAScript, defined by the ECMA-262 standard published by Ecma International [10]. So paradoxically, JavaScript led to the development of the ECMAScript standard that now governs the development of JavaScript. JavaScript also contains functionality to access technology standards not governed by Ecma International, such as HTML5 <canvas> [11] and Web Graphics Library (WebGL) [12] for 2D and 3D graphics in webpages.

JavaScript is not the only implementation of ECMAScript. Due to the large number of developers programming in JavaScript (and therefore ECMAScript), ECMAScript has been used as the framework to develop other, non-browser-based technologies. Node.js [13] compiles ECMAScript for server systems. ActionScript [14] was an Adobe implementation of ECMAScript providing scripting functionality to the now deprecated Adobe Flash Player platform. Rhino [15] and its replacement Nashorn engine [16], provide Java based ECMAScript scripting environments in Java applications. The multitude of ECMAScript implementations causes confusion as many engines based on ECMAScript are described as providing a JavaScript environment in their particular platform. This is false and due to name recognition. JavaScript is strictly the web based implementation of ECMAScript for internet browser engines.

While ECMAScript certainly has a range of utility and implementation across a variety of platforms, this review focuses on the JavaScript implementation of ECMAScript as it applies to client side HTML5.

### HTML5

The content we view on the Internet is made possible by a union of several technologies, mainly Hypertext Markup Language (HTML), Cascading Style Sheets (CSS) and JavaScript. HTML is an Extensible Markup Language (XML) protocol defining the Document Object Model (DOM). XML is a protocol defined by a clear and extensible syntax of elements and properties [17]. The DOM is an organization of the elements and properties defining the data for the page. CSS provides a powerful way to control the rendering properties of elements in the DOM, as well as selectors for efficiently styling classes and groups. JavaScript is the programming language that allows a developer to perform functions and interact with the DOM.

HTML5 is a moniker describing the most recent iteration of these internet technologies, namely it is the current version (version 5) of HTML. HTML4 defined most of the history of the Internet. HTML4 provided only limited functionality to website authors; therefore solutions like Flash and Java applets came into existence. HTML5 defines features previously only available in plug-ins, directly through native browser functionality while providing extensibility not possible with previous versions of HTML.

New web technologies emerged, such as native video support, localStorage, <canvas>, WebSockets and many more, allowing developers to fully support any application, natively in the browser. Access to these features needed to be facilitated, and the programming language to control all this additional functionality is JavaScript, the native browser language. JavaScript became much more powerful due to the evolution of HTML.

At the time of writing this review, HTML5 has had many years to mature. We now see HTML5 applications across every platform imaginable, fueled by the growth of mobile devices where internet browsers are first class citizens. It is this wealth of functionality, access and ease of distribution that has lead to JavaScript becoming an essential programming language for scientists and especially for chemists. Several large, open source, JavaScript chemistry libraries are actively maintained, including the ChemDoodle Web Components [18], Kekule.js [19] and 3Dmol.js [20], to support the chemistry community.

## Features

JavaScript is not developed with scientists in mind and is not, in any way, optimized for the sciences. The first high level programming language, Fortran [21] (“FORmula TRANslation”), was specifically designed to support advanced mathematical and scientific equations, and due to its performance, is still used today in many academic circles. Python [22] is a scripting language with an easy to learn syntax and core libraries dedicated to scientific computing. Yet, JavaScript exhibits a number of unique qualities greatly benefiting scientists. Development tools are available on every scientist’s computer through a web browser, free of charge. Distribution through the Internet is intrinsically supported. The free availability is essential to the core goals of science to distribute fact and information and to inspire and educate individuals to pursue knowledge. Regardless, there are a number of caveats to using JavaScript, and any scientific programmer should be aware of all of the technical features discussed below.

### Performance

The speed of calculations is a top priority when considering a programming language. The faster an application is, the more calculations it can compute and the more users it can serve. JavaScript performs on the slower end of programming languages. As a scripting language being run in a web browser, JavaScript cannot be faster than the platform it runs on, typically a C [23] derivative. The interpretation of JavaScript at runtime makes it inherently slower than its platform environment, even when taking advantage of just-in-time (JIT) compilation. Simple JavaScript applications can provide a quick and efficient interface, but will suffer as the complexity of calculations increases. Chemistry algorithms are especially sensitive, where molecules are represented as graph data structures. Runtimes for graph theoretical algorithms will scale exponentially as molecule size increases.

The "Performance results" section describes some tests to compare JavaScript and Java performance for cheminformatics tasks. Runtimes are clearly slower for JavaScript, but maybe acceptable if simple data is run in an efficient browser. However, as the data structures become more complex, the runtimes for JavaScript increase significantly and at a greater rate than Java. The results also highlight another issue: JavaScript performance varies widely between browsers, and this must be considered when creating any JavaScript application, as users will have their preferred browser. Extrapolating to the most expensive computational chemistry tasks, a fully ab initio quantum mechanics calculation would take days on a super computer, and would surely be wasted as a JavaScript implementation. However, there have been some implementations of intensive chemistry algorithms in JavaScript. Ertl et al. demonstrated a fully functional database with graph isomorphism searching directly in JavaScript [24], transpiled from OpenChemLib [25] (transpiled source code is automatically generated from source code in another programming language). The JavaScript cheminformatics library, Kekule.js, includes molecular geometry optimization features transpiled from OpenBabel [26]. GLmol has an extension allowing it to create full molecular surfaces for proteins based on the EDTSurf algorithm [27]. Not one performs at commercially acceptable speeds.

Poor performance is further compounded when trying to target mobile platforms, as mobile devices typically have less powerful hardware than traditional computers. This is a serious burden on the developer, as special care is needed when crafting JavaScript applications, and the efficiency of written code must always be scrutinized. Web sites and applications that are slow to load or execute will be immediately ignored by web surfers and penalized by search engines.

It is clear that native implementations will always have a significant performance advantage over JavaScript implementations. Regardless, there are encouraging trends as technology evolves: we continue to see more powerful processors on smaller devices, mitigating performance issues. Eventually, the slow JavaScript applications of today will be the fast applications of tomorrow. And for now, JavaScript’s poor performance is far outweighed by its ubiquity on all platforms.

### Distribution

When creating any application, a developer intends to reach as many users as possible. Developers therefore choose a programming language suited to the platforms presentable to the largest number of potential users. Because each Operating System (OS) is unique (Windows executables cannot be run natively on macOS, for instance), significant resources are required to port applications to each platform. If these resources are not available, the choice becomes which users to exclude. Fortunately, some programming languages were conceived to obviate that choice by providing a layer over the native platform of several operating systems, so code can be “written once and run everywhere” (coined by Sun Microsystems), thereby maximizing the developer’s effort. This was the inspiration to create the Java programming language; to allow developers to provide applications across Windows, macOS (formerly Macintosh and Mac OS X), Linux and others. Java remains a popular programming language today for producing scientific applications. Similarly, JavaScript became the programming language of the web; every significant web browser in existence, across traditional, mobile and emerging platforms, has built-in support for JavaScript. Many popular platforms, such as iOS, do not support Java, but do support JavaScript. In this way, JavaScript has surpassed Java as a universal programming language; no other programming language rivals JavaScript in its accessibility.

On mobile systems in particular, there is significant disagreement among developers about native implementations versus web based systems. Phones are technological Swiss army knives. Yet many of their features are not accessible in JavaScript (similar to how native desktop OS features are not always available in Java). To further capitalize on JavaScript work, systems like PhoneGap [28] and Cordova [29] use the native WebView components of the mobile operating systems to create native apps that can take advantage of features not available in mobile web browsers. WebView based solutions also provide a means for deployment through popular mobile app stores. Currently, a different approach for creating mobile apps using just web technologies called a Progressive Web Application (PWA) [30] is gaining popularity among developers. Such JavaScript implementations can help chemistry software developers avoid significant development costs for each platform.

Another reason JavaScript is easy to distribute is no pre-compilation is required, nor is the creation of an executable. It is important to understand, while a server is required to host and distribute web applications, as a scripting language, JavaScript source code is downloaded and interpreted at runtime. Therefore, licensing becomes very important as JavaScript source code is difficult to protect, and is very easy to view, understand, modify and copy. Owners of proprietary projects must keep this in mind. Open source projects may benefit from this as the code is readily accessible when used, but special care should be taken to understand one’s own licenses or the licenses associated with the JavaScript libraries being integrated. For instance, the commonly used General Public License (GPL) [31] will automatically become enforceable for JavaScript code as the mere act of accessing a web page running GPL licensed JavaScript code requires that code to be distributed to client machines.

Finally, due to the issues involved in distributing large JavaScript projects, several JavaScript source processors exist, including Google Closure Compiler [32], the Yahoo! User Interface (YUI) [33] library and UglifyJS [34]. These tools can minify and optimize source code by using certain patterns to shrink the size of JavaScript source downloads and optimize the code for more efficient performance (minification is a term specific to web technologies such as JavaScript, where small download sizes are optimal, and should not be confused with chemical applications of minimization, such as energy minimization or waste minimization). Such post-processing is strongly recommended when writing usable scientific programs. Some of these tools also have the ability to obfuscate JavaScript source, and when run completely, will destroy any intentionally public Application Programming Interface (API), but may help to protect proprietary code. It should be clear though, no amount of JavaScript obfuscation will be able to stop a determined hacker from reverse engineering or finding vulnerabilities in JavaScript work.

### Security

Aspects of the JavaScript programming language make security an important concern. Traditional applications run on the OS. Users will verify the legitimacy of the programs they use or rely on virus scans to protect their computers. JavaScript, however, is run on webpages just by visiting them. Given the vast number of users visiting web pages continuously, JavaScript is a prime vector for hackers to distribute harmful software. To reduce risk, JavaScript is sandboxed in the web browser and does not have access to the same features essential to other programming languages. There is no access to the file system or the system clipboard for copy and paste functionality. Browser producers may impose further restrictions, including ignoring running code attempting to identify and track users or disabling JavaScript based ads in web pages. Performance intensive applications may also be throttled by browsers. For example, there are limits to the resources available to WebGL canvases, and any long running algorithms may be halted.

The client side execution of JavaScript leaves it open to another attack vector. Since JavaScript is interpreted at runtime, any capable developer can remove, modify or inject their own behavior into web applications. This introduces special concerns for products enforcing integrity of data, such as eLearning systems. If grading mechanisms for such a system are exposed in JavaScript, a student can simply modify relevant code when running that application to achieve a perfect score without doing the exercises. System vulnerability can be compounded, as individuals without programming knowledge may naïvely rely on 3rd party hacks to control the JavaScript software, exposing them to malicious behavior including the wide-scale transmission and monitoring of information about the application and its users.

If any personal or proprietary data is processed in JavaScript, the data will have to be downloaded and accessible to the user, compromising any confidentiality. Regardless of the best practices a company should uphold, new laws are now ratified to protect user information. The European Union (EU) began to enforce the General Data Protection Regulation (GDPR) in 2018 [35], requiring all companies to gain consent for the acquisition and handling of user information. Handling of data in JavaScript is also troublesome for electronic laboratory notebooks, where 21 CFR Part 11 [36] compliance is required. So JavaScript applications need to be built in a way to protect the integrity of systems and the confidentiality of information. These considerations are essential, but inherently difficult, for small scientific startup companies.

There are some common practices a developer should follow when creating JavaScript applications. Hypertext Transfer Protocol encrypted using Transport Layer Security (HTTPS) [37] utilizes a Secure Sockets Layer (SSL) technology to encrypt communications between users and a server, protecting them from hackers and data snooping. Implementation of HTTPS requires significant technical knowledge to implement, and financial resources for the SSL certificate, but without it, users are left vulnerable. Any unprotected web application will, at best, be viewed suspiciously and will ultimately be penalized by search engines. Sensitive data should only be handled in JavaScript when absolutely necessary. Any user information, for instance, proprietary molecular data, should be encrypted and sent to a properly administered server for any storage or processing.

The accessibility of JavaScript applications is worthwhile to scientists, although security issues are a significant concern. Another powerful quality of JavaScript makes up for this weakness. As a web technology, JavaScript has built-in, real-time access to server resources, protecting user information and proprietary algorithms on an administered server. Any technology can then be distributed through a JavaScript graphical user interface (GUI). As a result, JavaScript possesses a unique ability for making even the most specific, hard to use, and unsupported scientific code bases accessible to users around the world. JavaScript truly facilitates collaboration and the dissemination of knowledge.

### Legacy code

As web applications grew, developers pursued creative ways to engage with users in JavaScript, the primary restriction being that content on a page was limited to what was downloaded. If content was to be changed, for example based on information in a server database, the web interface needed to communicate the changes to a server, then re-download the new content to be viewed, forcing the user to tolerate a reload of the web page. Microsoft introduced a new solution to this in 1999 with the XMLHTTP ActiveX control in Internet Explorer [38]. This new technique facilitated direct access to a server in JavaScript. As an asynchronous call, connections to the server would not block the responsiveness of the interface, and the direct communication of information back to the JavaScript call allowed the developer a powerful technique to interact with the user. All other browsers adopted this technique as the standard web technology, XMLHTTPRequest (XHR). Development patterns making use of the XHR object became known as Asynchronous JavaScript and XML (AJAX).

For security reasons, AJAX requests were limited to contacting the host origin, but HTML5 brought XHR2, which provided a new protocol to allow XMLHTTPRequest to contact and validate connections to external origins.

A system utilizing AJAX to communicate chemical information to the server can take advantage of any chemistry technology in existence. Even old legacy code can be hosted on a server and accessed with a JavaScript GUI. Proprietary code can be protected, and programs requiring advanced expertise and resources to compile and run can be deployed as a service. CPU intensive algorithms, such as energy minimization or quantum mechanics calculations, can be hosted on powerful servers, so users have an alternative to longer run times on their less powerful computer. Many chemistry databases are working to provide services through AJAX. One notable example is the Macromolecular Transmission Format (MMTF) [39] JavaScript libraries distributed by the Research Collaboratory for Structural Bioinformatics (RCSB), aimed to improve Protein Data Bank (PDB) handling in web applications.

Although XML is stated as part of the AJAX process, any protocol may be used to communicate with the server. Most applications use JavaScript Object Notation (JSON) [40], which is a protocol for defining data structures in JavaScript. JSON is a native feature in JavaScript syntax, so JSON in JavaScript source is easily written and efficiently processed. Several JSON formats were conceived specifically for chemistry. The ChemDoodle JSON format is designed as a minimalistic and extensible definition for molecules and their associated chemical and graphical objects and properties, both in 2D and 3D [41]. Both the open source ChemDoodle Web Components and OpenBabel provide support for ChemDoodle JSON. The OpenChemistry project [42] has also defined a Chemical JSON format [43]. OpenEye proposed a chemistry oriented JSON format with a focus on extensibility, but it has ceased to exist. Use of the browser *JSON.stringify()* and *JSON.parse()* functions allows the developer to convert between JavaScript and String objects, to facilitate JSON communication with a server through AJAX.

Another popular chemistry protocol for the web is Chemical Markup Langauge (CML) [44]. CML is an XML format, just like HTML, so CML can be embedded directly in the DOM, but it must be manually processed to be handled in JavaScript, unlike JSON formats. CML is a mature chemistry protocol with support in a number of toolkits, making it a prime choice for server side handling.

Another method to use legacy code bases in JavaScript is called transpilation, where source from another programming language is automatically converted into JavaScript source. The JavaScript vulnerabilities in performance and security remain. JSME [45] was transpiled from a Java applet to JavaScript with the use of the Google Web Toolkit (GWT) compiler [46]. Kekule.js developers use Emscripten [47] to port functionality from the C library, OpenBabel, to JavaScript. JSmol [48] is a transpilation of the Jmol Java applet into JavaScript making use of the SwingJS tool [49]. There are three considerations when using a transpilation tool: (1) the process will not be perfect, so the resulting code will have to be scrutinized, and likely post-edited, to ensure proper behavior; (2) the transpiled code may be injected with a middle layer introducing overhead in the application, compounding the performance issues in JavaScript possibly leading to the inconvenience of loading screens; (3) the resulting transpiled code, constructed by a machine, is difficult for the average developer to decipher. Transpiled source code defeats the purpose of open source software in JavaScript, as the original code base is necessary to make any changes, and the transpilation process must be run again to produce a JavaScript result. Proprietary code transpiled into JavaScript is inherently more prone to reverse engineering than the original source. Transpilation is best reserved for GUIs when the authors do not have the technical ability or opportunity to create a complementary JavaScript project.

Some JavaScript applications are impractical due to performance and security issues. Through servers, JavaScript developers can protect their systems and users and benefit from existing technology. This is very important for chemists, as there is a significant amount of computational innovation across many programming languages since the inception of computer systems. We want to retain access to the work of the computational chemists of the past. In the end, JavaScript applications excel at providing graphical user interfaces when paired with server side functionality.

### Graphics

Several steps were necessary to bring JavaScript to its current excellence in visualization. JavaScript originally only manipulated HTML DOM elements and CSS. So the early chemistry interfaces were limited to input forms allowing some calculation to be output. Further work allowed for the presentation of periodic tables. WebElements [50] and Ptable [51] are two of the earliest examples. Rendering of molecular structures was not practical with just the HTML elements available; Adam Grossman demonstrated a simplistic molecule viewer with just HTML elements and CSS [52].

The advent of HTML5 and the introduction of the <canvas> element enabled JavaScript to create graphics. With the <canvas> element, a JavaScript developer can draw 2D graphics in a DOM element. When text rendering capability was added to <canvas>, chemists had all the tools necessary for publication quality 2D graphics natively on the web. The ChemDoodle Web Components library [18] was the first JavaScript solution for rendering 2D chemical structures using <canvas>. Several tools attempted to use <canvas> to render orthographic 3D structures, including CanvasMol [53], ChemDoodle and TwirlyMol [54]. More complex PDB structures incorporating ribbon diagrams were successfully rendered by Jolecule [55] using just <canvas> graphics in 2D.

Introducing 3D hardware accelerated graphics as a JavaScript ability was the next step. WebGL was introduced to provide JavaScript bindings for OpenGL, specifically OpenGL ES 2, through the <canvas> element. The ChemDoodle Web Components library was again the first toolkit to provide 3D graphics of molecules using WebGL [56]. SpiderGL [57] was also documented. GLmol [58], and associated forks (a fork is a copy of the original project where new developers typically work towards a different goal), are based on the three.js WebGL library [59].

Scalable Vector Graphics (SVG) [60] is a complementary graphical feature in HTML5. SVG is an XML protocol defining 2D drawing instructions for graphics, but lacks capability for defining 3D objects. HTML5 <canvas> is based on a bitmap buffer, so the scale and resolution of the screen or device must be properly taken into account for clear graphics. SVG rendering is independent of the resolution or scale of the device. Creating dynamic applications with SVG is more difficult as any changes require DOM manipulation.

The most important interface enabled by these graphical features is a chemical sketcher. Chemistry is unique because a molecular structure is the core unit to be communicated. The browser natively supports other interfaces through forms containing text fields and check boxes and calendars, but lacks a chemical sketcher. The availability of a JavaScript based chemical sketcher is therefore essential to chemists. The first example of a chemical sketcher in JavaScript was jsMolEditor [61]. Currently, advanced, open source, chemical drawing interfaces exist in pure JavaScript, including the ChemDoodle sketcher [62], Ketcher [63] and in Kekule.js.

One serious topic involving interfaces is accessibility for individuals with disabilities. Chemistry software developers should concern themselves with making their work accessible. HTML5 introduced features for controlling graphics and sound, and touch screens allow many creative input solutions through software and hardware accessories. JavaScript is present on these emerging platforms, with APIs to work with traditional mouse and keyboard events as well as touchscreen events and gestures. This is beneficial for organizations creating educational products as the Americans with Disabilities Act (ADA) requires accessibility considerations in chemistry software in the United States [64]. Many governing bodies enforce accessibility in public software as well, adhering to the Web Content Accessibility Guidelines (WCAG) specification [65], which is an International Organization for Standardization (ISO) standard. Through JavaScript, the communication of chemistry between individuals and computers has never been easier.

Chemical applications on the web greatly expanded with the capability of communicating chemistry in both 2D and 3D in JavaScript. Currently, almost every major chemistry related software product has integrated a web based interface using JavaScript. Two notable examples are SciFinder, and a new solution from the Chemical Abstracts Service (CAS), SciFinder-n [66], using JavaScript interfaces to provide further access. SciFinder presents the venerable CAS content collection, a clear validation of the importance of JavaScript interfaces in commercial chemistry products.

### Further innovation

Many new JavaScript features continue to be conceived. A feature called localStorage (and its companion sessionStorage) allows JavaScript applications to store information through page loads [67]; Web Workers provides a framework for multi-threaded JavaScript applications [68]; the <audio> tag allows sounds to be played in JavaScript [69]; WebSockets allows a continuous link between a JavaScript application and a web server for direct and constant communication [70]. Continued innovation in JavaScript is a direct result of developers’ work to provide solutions on the web.

## Programming

JavaScript exhibits the common aspects of most programming languages. Semantics and a syntax familiar to programmers are defined to communicate with the computer. There are primitives, functions and for loops. JavaScript also harbors a number of significant differences including its scopes and context, the global namespace and the need for browser standardization. In this section, an outline of important topics for programming chemistry in JavaScript is presented. Many resources exist to introduce those interested in computer science to programming. JavaScript is a web technology, and the vast majority of instructional material for JavaScript can be found freely on the Internet. We will be focusing on the technicalities of JavaScript programming. The instruction begins with a reinforcement of JavaScript basics to prepare readers for more complex constructs, leading to the implementation of Object Oriented Programming (OOP) in JavaScript and how chemistry can be represented.

At the time of this writing, the latest version of ECMAScript is version 9, also known as ECMAScript 2018 [10]. Technologies evolve rapidly and information herein may become obsolete as changes are made.

### Reinforcing the basics

JavaScript is an easy language to learn, but a very difficult one to master. Additionally, certain programming constructs can be coded using many different syntactical approaches, making the language very inconsistent. Yet, this lack of rigidity makes the language very malleable, and the possibilities are only restricted by the creativity of the developer. Developers attempting to create the complex applications necessary for chemistry without fully understanding the unique aspects of the language will encounter serious issues. Most importantly, an understanding of scope, context and closures is necessary to properly direct the behavior of JavaScript applications. These topics are covered in more detail in the following sections.

Several source code listings accompany the following topics. There are several ways to execute JavaScript code. The first is through a browser’s JavaScript console. The second is by including JavaScript directly in the DOM for a loaded web page using <script> tags. The third is through the import of a JavaScript source file in a web page, typically with a .js extension.

#### Integrated Development Environment

An Integrated Development Environment (IDE) is a powerful tool for developers to quickly address mistakes and errors when writing code. IDEs are less practical for JavaScript. While several IDEs exist, core JavaScript behavior does not come from a standard compiler, but through each of the individual browsers. So no IDE can truly provide an accurate development environment. It is often best to write JavaScript code and then test it in each browser being targeted, typically all of them.

Fortunately, each browser contains a JavaScript console for examining errors, running snippets of code, altering interpreted code and more. For instance, in Chrome, we can access the JavaScript console by selecting the **View>Developer>JavaScript Console** menu item. Figure shows the JavaScript console in Google Chrome.
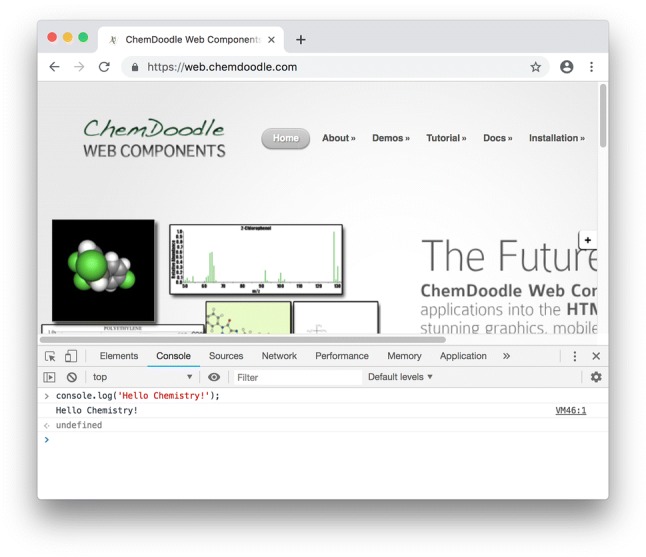


We can output to the JavaScript console using the *console.log()* function as shown in Listing 1. The JavaScript interpreter will ignore comments in source code, designated as any text on a line preceded by a pair of forward slashes (//).

**Listing 1:**
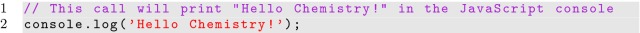
Using the console.log() function

Note that JavaScript strings should typically be declared with single quotes. There is no difference between a JavaScript string created with single quotes and one created with double quotes. However, JavaScript works in an HTML environment where any XML strings will undoubtedly contain double quotes. Using single quotes allows the quick writing of strings with double quotes included, without having to escape them or deal with difficult to find string construction errors.

#### Browser standardization

One of the significant drawbacks to creating JavaScript applications is the lack of a standard compiler. ECMAScript defines the JavaScript language; implementation is left up to each browser. This fragments the web ecosystem and developers would be unaware of any issues in the browsers they do not use. Fortunately, there are well supported JavaScript projects aiming to standardize JavaScript behavior across browsers. jQuery [71] and Sencha [72] are popular examples. These libraries were essential in the past, as huge discrepancies between JavaScript implementations existed, but are becoming less important as modern browsers work to achieve a more consistent implementation. Reliance on browser standardization libraries should only be allowed when code maintenance is improved, such as with the DOM manipulation features of jQuery.

As HTML5 continues to evolve, new features continue to be invented and extensions to old features are introduced. Each of the browsers is developed independently on its own schedule, so implementation of HTML5 features is far from synchronized. Polyfills can be used to inject behavior before features are natively implemented, and removed when the features are universally available. For instance, *requestAnimationFrame()* is recommended for WebGL animations, while *setTimeout()* is the traditional function for working with timers. A polyfill can check if *requestAnimationFrame()* exists, and if not, create one using the *setTimeout()* function.

#### Variable typing

JavaScript is an untyped programming language (also referred to as weakly typed), which means the interpreter will not care what type of data is defined to a variable until evaluating an expression. A variable can be declared as a number, and then later set to a string. The freedom an untyped programming language provides is very powerful, but it is necessary for algorithm logic to be aware of the variable types, and to avoid changing them, as the interpreter will automatically cast mixed types to the same type for execution, leading to unexpected results or errors.

To overcome some issues with variable typing, JavaScript includes a pair of strict equality operators, *===* and *!==*, in addition to the typical equality operators, *==* and *!=*. The typical operators will match values even if the variable types are different, for instance the number 10 will match the string ‘10’, and the number 0 will match the Boolean false. The strict operators not only check for value equivalence, but also that the types of values on both sides of the operator are equivalent. The strict equality operators are therefore less error prone and should always be preferred in application logic.

#### Declaring variables

Originally, a variable could be created in one of two ways in JavaScript, without a keyword or with the *var* keyword. ECMAScript 6 introduced two new ways of declaring variables using the *let* and *const* keywords.

The *var* and *let* keywords are very similar, and most well written code would not see any difference in behavior when switching between the declarations. Technically, *var* binds a variable’s visibility to the closest enclosing function or global scope, while *let* binds a variable’s visibility to the closest enclosing block or global scope. The differences between the behavior of the *var* and *let* declarations can be seen in Listings 2 and 3. A variable can be accessed before it is first declared with the *var* keyword; this is referred to as variable hoisting, and can cause errors if not properly understood. Using *let* will help to avoid programming errors if the same variable name is accidentally declared twice in the same scope since an error will be thrown. Using *let* also avoids semantics issues when multiple scopes have access to the same variable. This has important ramifications, especially when utilizing the for loop variable to generate functions, which are common when building JavaScript interfaces.

**Listing 2:**
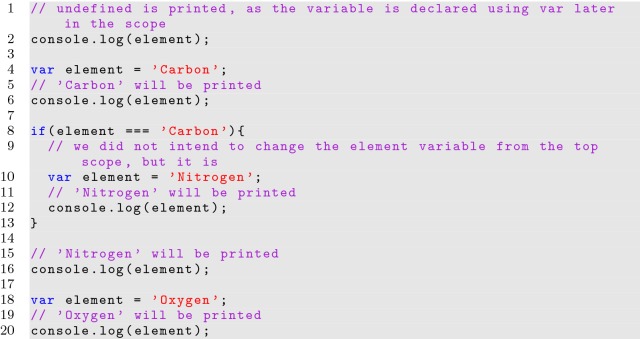
Using the var keyword

**Listing 3:**
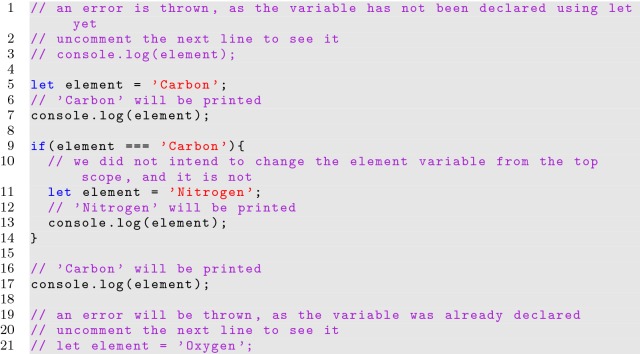
Using the let keyword

Additionally, using *let* provides more control in the global scope, as such declared variables will not be defined to the *window* object. The *window* object is the standard, top-level, JavaScript object. While older code will typically use the *var* keyword as it was the only option, *let* should now always be used instead.

The *const* keyword declares an unchangeable variable, similar to the *final* modifier in Java. If a *const* variable is assigned to an object, the object’s properties can be changed, while the variable pointer to the object cannot. This technique is useful when creating a value that should remain consistent throughout the lifetime of the application, such as core bond order variables in cheminformatics toolkits.

If no keyword is used when declaring a variable, the visibility of the variable becomes global regardless of the scope it is in, polluting the global namespace and potentially causing issues. This should never be done.

#### Lexical scope

JavaScript is meant to be executed for a web page, and source can be interpreted at any point for a page, before or after the DOM is closed. So unlike traditional application source code that is compiled and run, JavaScript code is run by loading a web page into the browser.

Due to the need for JavaScript to access all the aspects of a web page, the main programming construct is basically a giant resource pool. This pool is specifically called the global scope and the union of all variables defined in the global scope is called the global namespace. Lexical scope in JavaScript is therefore more complex than in other languages. Everything and anything pertaining to the webpage can be accessed in JavaScript through the global scope, exposing implementation and behavior to other parts of the application, other developers and even users during runtime. JavaScript resources do not persist between page loads (an exception is the *window.name* variable as well as *localStorage* and *sessionStorage*).

When a function is defined, a new scope is produced, denoted by a pair of enclosing curly braces. This is called a local scope. All scopes have access to the variables contained within and in parent scopes up to the global scope, but do not have access to variables in their child scopes. The combination of a function with its own scope and all of the variables the function has access is known in JavaScript as a closure. Closures are an important concept in JavaScript. When only using the *let* variable declaration, any statement block curly brace pairs will define a local scope, not just functions.

In addition to exposing implementation, the global scope can become a hindrance to programming, as care is needed to avoid name clashes that would overwrite previously defined variables. As multiple libraries are included into a webpage, such conflicts are inevitable. In chemistry, every library will undoubtedly contain a “Molecule” class. The increased probability of conflict caused by creating global variables is called global namespace pollution. Avoiding pollution is a requirement for any usable scientific library. Techniques for doing so are discussed in the "Object Oriented Programming" section.

#### Undefined values

It is also important to represent values that have not yet been set, and JavaScript has two keywords for this: *undefined* and *null*. This can lead to confusion, as many programmers are familiar with the *null* keyword from other languages. In JavaScript, *undefined* refers to a variable that has been declared, but not assigned, while *null* is actually an object used by a developer to represent nothing. Therefore, the *null* keyword would only be useful for specific logic as it must be assigned, and is never necessary. The *undefined* keyword should always be used to check for unassigned variables as shown in Listing 4.

**Listing 4:**
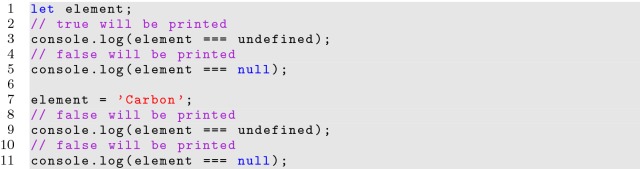
Behavior of undefined and null

#### Creating objects

Objects can be created in JavaScript by assigning a variable to curly brace pairs as shown in Listing 5. An array (which is special object) can be declared similarly, but with square bracket pairs. Notice we can use JSON to fully define object parameters. Object properties, including functions, can be redefined at any point during runtime.

**Listing 5:**
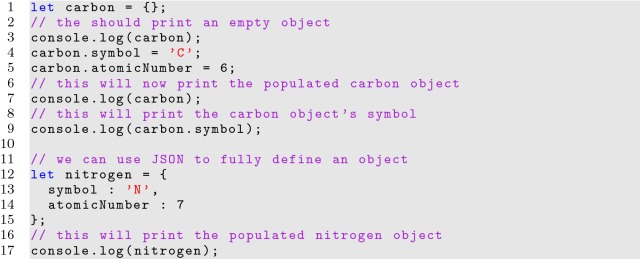
Creating an object

#### Creating functions

Functions also have unique behaviors in JavaScript, as functions are first class objects and can be assigned properties. Listing 6 shows how we create a function.

**Listing 6:**
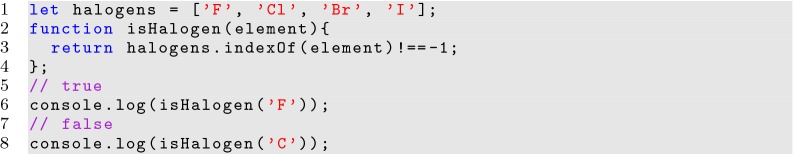
A function declaration

The created function is now an object in its enclosing scope. The function can be accessed as an object by using the function name and can be executed by using the function name followed by an invoking pair of parentheses.

Notice the included semicolons after every declaration, even for functions as in line 4 of Listing 6. While semicolons are not required in JavaScript, including them makes code easier to read, helps the interpreter and will remove any ambiguities that may arise.

JavaScript primitives perform in a similar manner to primitives in other programming languages. JavaScript primitives are passed as values when included as a parameter in a function, while objects are passed as pointers, which means manipulation of an object inside of a function will change the original object used to call the function. JavaScript functions can return data, as in other languages, but if no return statement is included in a function, *undefined* will be automatically returned upon completion.

We can alternatively create the function with a more obvious object syntax as shown in Listing 7. Any data is assigned to the function to avoid polluting the global namespace. The method of creating a function shown in Listing 6 is called a function declaration, while Listing 7 uses a function expression. Only function declarations are subject to hoisting by the JavaScript interpreter and will be available at any time in its enclosing scope.

**Listing 7:**
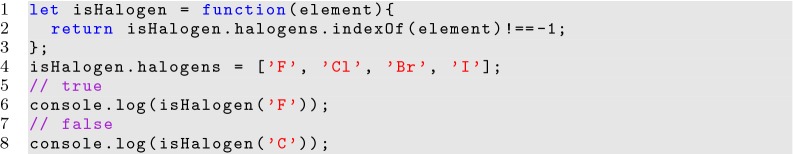
A function expression

Functions form the basis for class creation in JavaScript, which will lead to the implementation of OOP; this method of programming is essential for implementing chemistry algorithms.

### Object Oriented Programming

In contrast to procedural programming, OOP enforces a data structure centric logic for software development. Such a paradigm produces code that is easier to read, compartmentalized, reusable and less prone to errors. This model is particularly beneficial for chemistry applications, as molecules are represented as graph data structures. When properly implemented in JavaScript, OOP APIs benefit from protection of implementation details and a reduction in global namespace pollution. Many resources exist for introducing OOP to the interested developer. The following section discusses the implementation of OOP in JavaScript.

#### Classes

Object oriented programs build consistent instantiations of objects from defined classes. An object is programmatically instantiated from a class definition by invoking the class constructor. In JavaScript, a class constructor is represented as a basic function as shown in Listing 8.

**Listing 8:**
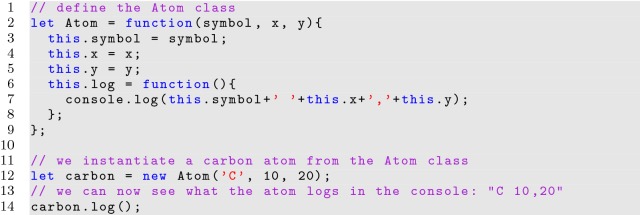
A basic class using a function expression

The *new* keyword is used to instantiate an object from the class definition as shown on line 12 of Listing 8. Once instantiated, associated class functions are accessible from the object. Functions declared inside of the constructor are called privileged functions and can access private variables defined in the constructor scope, but will be created anew for each instance.

JavaScript classes are not traditional classes, as would be found in OOP languages like Java and C++, which provide strict mechanisms for defining and extending classes. Instead, JavaScript uses the *prototype* keyword to describe inheritable properties for objects as shown in Listing 9. Functions set to the prototype are only created once for all instances. As a result, prototype functions are more efficient than privileged functions.

**Listing 9:**
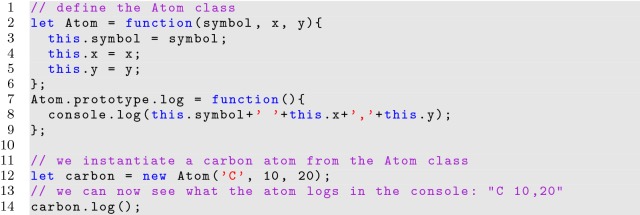
Defining a class using prototype

One of the most important aspects of OOP is extending classes. A child of the *Atom* class, called *Atom3D*, implementing a *z*-coordinate, is created in Listing 10. Checking class types in JavaScript is possible with the *instanceof* operator in expressions.

**Listing 10:**
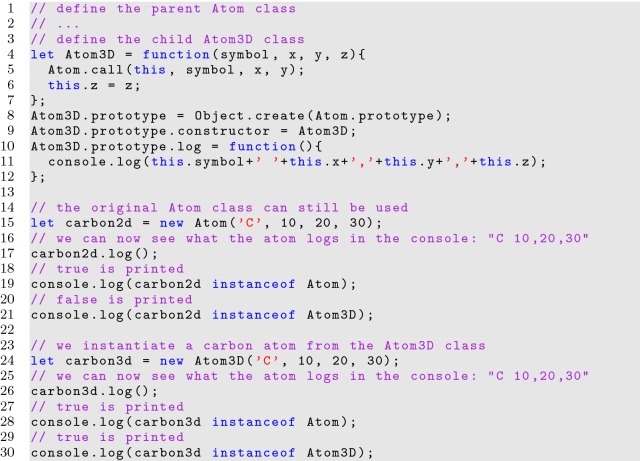
Extending a class

The prototype system in JavaScript facilitates extending parent class functionality. When an instantiated object calls a function, the interpreter first checks the object’s parameters in search of the function. If it cannot find the function, then the prototype’s parameters (through the *__ proto__* parameter) are checked, and then the prototype’s prototype, and so forth until the core *Object* prototype is reached. Functions defined to the prototype all point to a single instantiation, so at any time, the behavior can be changed for all instances of the class at once. Prototype based classes are therefore very powerful, and allow malleability not possible in traditional OOP languages because classes would be defined at compile time and unmodifiable.

ECMAScript 6 added traditional *class* syntax. While classes written this way will be more familiar to developers of other OOP languages, the underlying implementation in JavaScript still relies on the prototype system. Therefore, the JavaScript *class* syntax should be avoided, as it implies behavior not consistent with the way prototype classes work.

#### Context

Another peculiarity of JavaScript behavior is context, due to the unique scoping system. Context concerns what the *this* keyword references. Typically, in JavaScript, the *this* keyword allows programmatic access to the object performing the function, similar to standard OOP languages. So a created object will have the *this* keyword referencing itself and in any owned functions. Similarly, if the *new* keyword is used to instantiate a class object, all of the instantiated object’s functions will be able to access the instance they are bound to through the *this* keyword.

In the global namespace, *this* refers to the *window* object. In any functions created outside of objects, *this* also refers to the *window* object, unless the source is being interpreted in strict mode, *vide infra*, in which case *this* is undefined. There is an exception if the function acts as an event handler set through the *addEventListener()* function or through an inline on-event handler, and in these cases *this* refers to the DOM object firing the event.

There are also methods for overriding the reference of *this* in JavaScript. ECMAScript 3 added the *call()* and *apply()* functions for defining what the context, and therefore the *this* keyword, refers to during the immediate execution of a function. ECMAScript 5 added the *bind()* function to explicitly set the context for a given function regardless of how it is called.

#### Immediately invoked function expression

The openness of JavaScript resources through the global scope introduces programming issues for developers. An interesting JavaScript quirk can be used to provide a cleaner solution utilizing the grouping operator. The grouping operator should be familiar to all developers, as it is represented by a pair of parentheses in expressions to denote execution order precedence.

Programming language compilers typically implement the grouping operator by creating an unnamed temporary variable in the execution of an expression, otherwise known as an anonymous variable. JavaScript allows functions in expressions, and when a grouping operator surrounds a single function expression, the function itself is encapsulated as an anonymous variable. Therefore, source can be interpreted and executed without producing anything directly accessible in the current scope’s namespace, and hiding any internal variables from the outer scope, in essence, creating an anonymous closure. The anonymous function can then be invoked with a subsequent pair of parentheses. Such a construct is called an immediately invoked function expression (IIFE). An example is shown in Listing 11.

**Listing 11:**
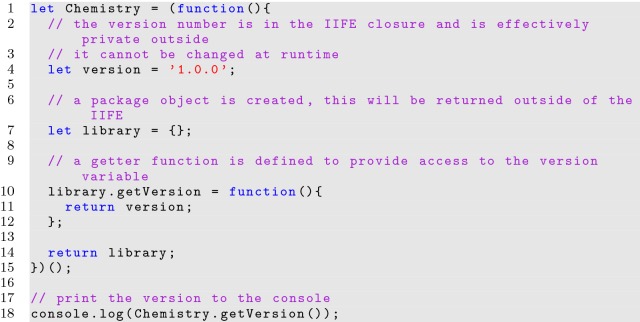
Implementing a library package using an IIFE

The final pair of parentheses used for invoking the function can be included inside or outside of the closing parenthesis of the grouping operator; its positioning makes no difference to the behavior.

IIFEs are incredibly useful in JavaScript programs, as such constructs produce a closure where variable visibility can be restricted, and the only variables set to outer scopes are what the developer intends to provide access to. In Listing 11, developers cannot modify the *version* variable at runtime, protecting internal implementation details relying on the variable, while still providing read-only access to the version through the lone *Chemistry* variable defined to the global namespace (a credible scientific library should always include programmatic read-only access to its version). This technique can be extrapolated to entire class definitions. Listing 12 shows the *Atom* class in an IIFE. Notice how the *isAllowedSymbol()* function is local to the IIFE and cannot be changed without modifying the source directly before interpretation. The developer wrote the function to facilitate functionality in the class, but does not want the function to be visible to others at runtime. Due to the local variables encouraged by IIFEs, JavaScript source code processors can be even more efficient at minifying source.

**Listing 12:**
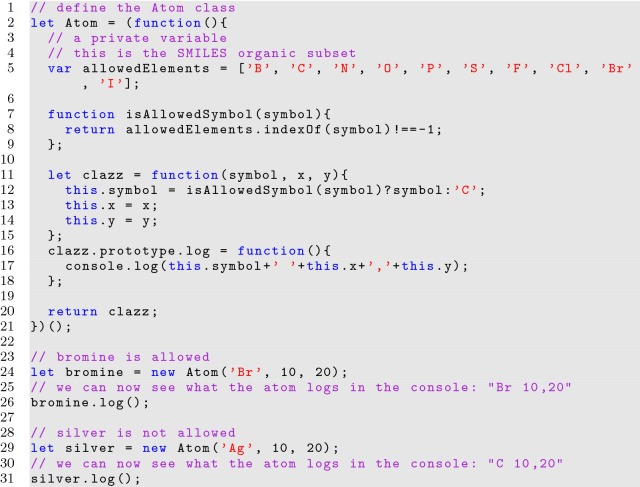
Privatizing variables in a class within a module

Many issues arise from the uniqueness of the JavaScript programming language. Cheminformatics applications in JavaScript can suffer if aspects of the code are exposed, given the complexity of chemistry based data structures and algorithms. JavaScript developers can address these issues by expertly working with the behavior of JavaScript interpreters and using IIFEs. IIFEs form the groundwork for building large and complex programs in JavaScript by giving developers control over the visibility of the components of an application.

#### Module pattern

Taking a step further, using IIFEs to create organized object oriented code is modeled by the module design pattern [73]. An IIFE facilitates OOP by providing a means to encapsulate JavaScript code, controlling implementation visibility while the module pattern organizes classes and functionality. IIFEs allow the simulation of an import system by passing variables through the invoking pair of parentheses. Listing 13 shows the import of the *console* global variable. Execution is now more efficient as the interpreter does not need to search for the *console* variable up through the global scope. Source code processors can further minify the IIFE source, as the *console* variable is now a local parameter. The *undefined* keyword is provided to the IIFE as the last parameter, even though it is not present in the invoking pair of parentheses. The additional parameter is provided because *undefined* can be declared as a variable name in JavaScript, and locally overriding the *undefined* variable to something that hasn’t been declared protects code logic from interference.

**Listing 13:**
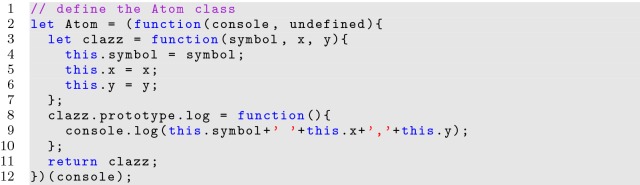
Using imports in a module

A library written in a single IIFE would quickly become unmanageable, and so several IIFEs can be used to link individual segments of logic, referred to as modules, into a single library utilizing parameter imports. Listing 14 shows how the module pattern can be used to organize discrete classes. Classes can then be easily added and removed.

**Listing 14:**
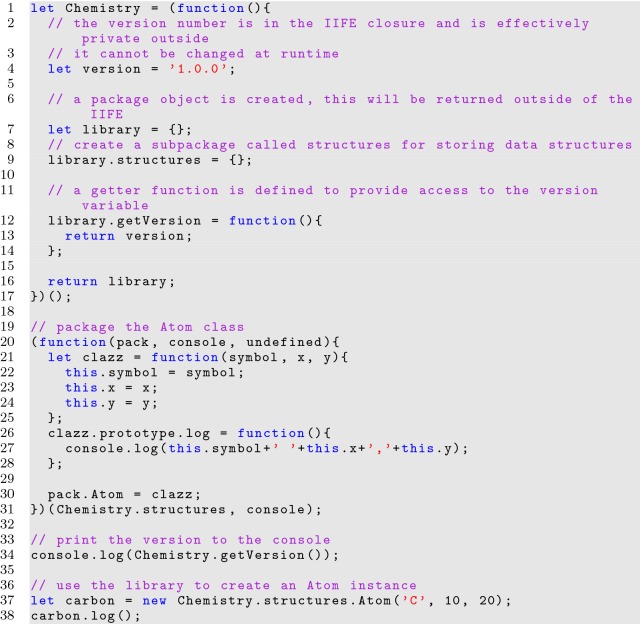
Creating a linked library with IIFEs

The module design pattern aids developers in writing more organized source code, where individual classes can be independent, allowing for extensible and sustainable libraries. By creating a linked library based on IIFEs, controlled access can be provided through a single variable placed in the global namespace. This is the best practice for avoiding global namespace pollution. While development is facilitated by this pattern, care must be taken when considering the dependencies linked into each module and their order in source.

Since modules are discrete segments of code, many developers separate modules into individual files that can be organized, reused and included in applications only when needed. The separation of modules into files results in the ability to build JavaScript files into custom libraries. To aid in the packaging of large JavaScript libraries, many tools exist to work with JavaScript module files, including Browserify [74] and webpack [75]. The module pattern is so influential that ECMAScript 6 added core module file support allowing functionality to be better organized with a defined *export* and *import* syntax, thereby standardizing how complex module-based libraries are declared.

#### Strict mode

Strict mode was introduced in ECMAScript 5 and allows the interpreter to reduce its flexibility in handling JavaScript source, so interpretation can be more clearly performed and less error prone. Developers must abide by additional restrictions when writing JavaScript code in strict mode. As a result, optimizations can be employed by the interpreter that would not be successful when interpreting random JavaScript source. Strict mode is initiated for the global scope or a function scope by including the *’use strict’;* phrase at the top of the scope as shown in Listing 15.

**Listing 15:**
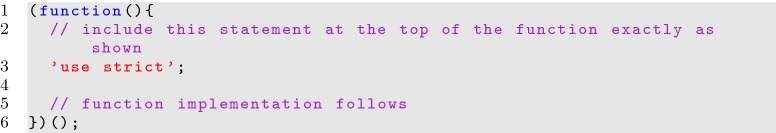
Using strict mode in a module

While strict mode is an important technique for writing better JavaScript applications, third party libraries should be integrated carefully in strict mode scopes as those libraries may not be compatible with strict mode.

## Performance results

Table 1 compares runtimes for reading the PDB entry 3CC2 into a data structure in memory from a string. Total times consist of 10 consecutive runs, after a 10 run warm up period is ignored. Bond deduction was disabled for both languages. Table 2 compares runtimes for perceiving the Euler facet ring set for C60 fullerene, while Table 3 collects runtimes for perceiving the Euler facet ring set for the unit cell of the LTA zeolite ignoring periodicity. Total times consist of 1000 consecutive runs, after a 100 run warm up period is ignored. The graph reduction step of the algorithm was removed in both languages, as the Java algorithm created a copy of the data structure, while JavaScript did not, leading to a biased overhead. The ring search algorithm was allowed to run to completion without any cutoffs. All tests were performed on a 2017 iMac running macOS 10.13.6 with a 4.2 GHz Intel Core i7. JavaScript tests were performed in Google Chrome Version 68.0.3440.84, Apple Safari Version 11.1.2 (13605.3.8) and Mozilla Firefox 61.0.1. The ChemDoodle Java API v2.4.1 [76] and the JavaScript ChemDoodle Web Components v8.0.0 were used, where the algorithms in both libraries were written by the same individual. Each test was run 5 times, with the fastest time recorded.

**Table 1:** Reading the PDB entry 3CC2 into a data structure in memory from a string

	Runtime (ms)
Java	795
JavaScript (Google Chrome)	1415
JavaScript (Mozilla Firefox)	1214
JavaScript (Apple Safari)	1394

**Table 2:** Runtimes for perceiving the Euler facet ring set for C60 fullerene

	Runtime (ms)
Java	1035
JavaScript (Google Chrome)	1134
JavaScript (Mozilla Firefox)	1379
JavaScript (Apple Safari)	8833

**Table 3:** Runtimes for perceiving the Euler facet ring set for the unit cell of the LTA zeolite ignoring periodicity

	Runtime (ms)
Java	3484
JavaScript (Google Chrome)	6689
JavaScript (Mozilla Firefox)	11960
JavaScript (Apple Safari)	53458

## Summary

At over two decades old, JavaScript is far from the end of its life, rather it seems like it is just beginning. The advent of the Internet not only connected us, but became an essential component of any device, leading to advanced browser support and therefore JavaScript support on any platform existing today. While JavaScript exhibits many weaknesses, its strengths are paramount, allowing not just the creation of pedestrian web based solutions, but in specifically providing a means for communicating the complexity of chemistry. As an open and standardized language, JavaScript has continued to thrive and evolve, while remaining a reliable foundation for developers. Scientists continue to find better and more powerful ways to use web technologies in the pursuit of science and to make knowledge accessible around the world. We will undoubtedly see continued technological innovation, and JavaScript, as the internet browser programming language, will likely continue to be the tool of choice for web developers and essential for the propagation of scientific information.

## Abbreviations

2D: two dimensional
3D: three dimensional
ADA: Americans with Disabilities Act
AJAX: Asynchronous JavaScript and XML
API: Application Programming Interface
AR: Augmented Reality
CAS: Chemical Abstracts Service
CFR: Code of Federal Regulations
CML: Chemical Markup Langauge
CSS: Cascading Style Sheets
DOM: Document Object Model
ECMA: European Computer Manufacturers Association
EDT: Euclidean Distance Transform
eLearning: Electronic Learning
ELN: Electronic Laboratory Notebook
EU: European Union
GDPR: General Data Protection Regulation
GNU: GNU’s Not Unix!
GWT: Google Web Toolkit
GPL: General Public License
GUI: Graphical User Interface
IDE: Integrated Development Environment
IE: Internet Explorer
IIFE: Immediately Invoked Function Expression
ISO: International Organization for Standardization
JIT: just-in-time
JS: JavaScript
JSON: JavaScript Object Notation
HTML: Hypertext Markup Language
HTML5: Version 5 of HTML
HTTPS: Hypertext Transfer Protocol encrypted using Transport Layer Security
MMTF: Macromolecular Transmission Format
MS: Microsoft
OOP: Object Oriented Programming
OpenGL: Open Graphics Library
OpenGL ES: OpenGL for Embedded Systems
OS: Operating System
PDB: Protein Data Bank
PWA: Progressive Web Application
RCSB: Research Collaboratory for Structural Bioinformatics
SSL: Secure Sockets Layer
SVG: Scalable Vector Graphics
WCAG: Web Content Accessibility Guidelines
WebGL: Web Graphics Library
XHR: XMLHTTPRequest
XML: Extensible Markup Language
YUI: Yahoo! User Interface
